# The Determination of Genetic Markers of Age-Related Cancer Pathologies in Populations from Kazakhstan

**DOI:** 10.3389/fgene.2013.00070

**Published:** 2013-05-02

**Authors:** Leyla B. Djansugurova, Anastassiya V. Perfilyeva, Gulnur S. Zhunusova, Kira B. Djantaeva, Olzhas A. Iksan, Elmira M. Khussainova

**Affiliations:** ^1^Laboratory of Molecular Genetics, Institute of General Genetics and CytologyAlmaty, Republic of Kazakhstan

**Keywords:** age-related disease, cervical cancer, esophageal cancer, genetic susceptibility, single nucleotide polymorphism

## Abstract

Aging associates with a variety of pathological conditions such as cancer, cardiovascular, neurodegenerative, autoimmune diseases, and metabolic disorders. The oncogenic alterations overlap frequently with the genes linked to aging. Here, we show that several aging related genes may serve as the genetic risk factors for cervical and esophagus cancers. In our study, we analyzed samples obtained from 115 patients with esophageal and 207 patients with cervical cancer. The control groups were selected to match the ethnicity and age of cancer patients. We examined the genes involved in the processes of xenobiotics detoxification (*GSTM1* and *GSTT1*), DNA repair (*XRCC1* and *XRCC3*), and cell cycle regulation and apoptosis (*CCND1* and *TP53*). Our study revealed that deletions of *GSTT1* and *GSTM1* genes or the distinct point mutations of *XRCC1* gene are associated with cervical and esophageal cancers. These results will lead to development of screening for detection of individuals susceptible to esophageal and cervical cancers. Introduction of the screening programs will allow the early and effective preventive measures that will reduce cancer incidence and mortality in Kazakhstan.

## Introduction

The aging of human populations is the most significant demographic change of the twentieth century. Despite the fact, that the population aging is particularly noticeable in the industrialized countries, a rapid increase of elder people is expected in populations of developing countries. Kazakhstan is one of the states with accelerated speed of aging. According to the United Nations data, every fourth person in Kazakhstan will represent the older population by 2050.

Aging is a complex biological process determined by genetic and environmental factors. Aging processes are associated with the accumulation of toxic metabolites, damages of biologically important molecules, and increased predisposition to the development of a number of pathological conditions. Age-related pathologies include cancer, cardiovascular, neurodegenerative, autoimmune diseases, diabetes, obesity, and other. It is known that the mode and speed of aging vary among different people due to individual genetic characteristics (Wheeler and Kim, 2011). The important areas of aging medicine are the elucidation of genetic and molecular mechanisms of aging including the role of genetic and epigenetic factors in the etiology and pathogenesis of various age-related pathologies. The development of age-related diseases or the ability to take an active longevity is greatly influenced by ethnicity (Gavrilov and Heuveline, 2003). A large number of centenarians are known for some ethnic groups, for example, Caucasian (Caucasian is also used for white-skinned people) people. The determination of genetic features of centenarians and genetic status of key genes involved in the pathogenesis of age-related pathologies represent the main approaches to define the key components of active aging and longevity.

Candidate genes participating in aging can be classified as following: (1) genes involved in tissue homeostasis (apoptosis and telomerase); (2) genes controlling integrity of genome and DNA repair; (3) genes involved in stress resistance (heat shock and oxidation); (4) genes contributing epigenetic changes (methylation, carbonylation, and nitrosylation).

Researchers are mainly focused on the genes whose orthologs determine longevity in other species and also on the genes responsible for development of the main age-related disorders.

Cancer is primarily a disease of older people where incidence rates increased substantially with age for most cancers. The genetic components of cancer overlays the range of candidate genes controlling aging. During the multistage carcinogenesis process, the cells predisposed to cancer accumulate mutations of proto-oncogenes, tumor suppressor genes, and other genes that are directly or indirectly involved in regulation of cell proliferation, survival, and migration.

Mutations of the same gene may cause the several cancer types. Thus, mutations of tumor suppressor gene *TP53* were detected in the tumors of all tissues and organs. The spectrum of mutations in key genes involved in the control of genome instability, DNA repair, cell cycle, apoptosis, and such processes as xenobiotics detoxification may vary for different cancer types.

As a result of natural selection the genetic polymorphism spectrum depends on the geographical conditions, diet, and ethnicity. In certain circumstances, genetic polymorphisms can predispose to the development of specific diseases, or to protect organism. Analysis of genomic polymorphism, which forms the basis of predictive medicine, helps to identify the individual genotypes that predispose to the development of diseases (Baranov, 2009; Yuzhalin and Kutikhin, 2012).

Here, we present the results of molecular epidemiological study of populations from Kazakhstan representing healthy individuals and patients with esophageal and cervical cancers. These cancer types have been selected in our study because of their high morbidity and mortality in Kazakhstan. Esophageal cancer is one of the most aggressive forms of cancer. It is ranked on the ninth place by malignancy and on the seventh place by mortality. Esophageal cancer often diagnosed at an advanced stage, and therefore the 5-year survival rate for this type of cancer is only in a range of 5–10%. The incidence of esophageal cancer in males reaches 25.7 cases in population of 100,000.

Cervical cancer in women is diagnosed in the reproductive age. Kazakhstan is among the countries with high levels of cervical cancer and its incidence is on the second place following the breast cancer.

Identification of genetic markers for these types of cancer will help to determine strategies of prevention, early diagnosis, and personalized treatment.

The choice of candidate genes for our study has been selected based on the previous studies (Tan et al., 2000; Gao et al., 2002; Dumont et al., 2003; Zhang et al., 2003, 2012; Abbas et al., 2004; Lu et al., 2006; Cescon et al., 2009; Francisco et al., 2010; Barbisan et al., 2011; Liu and Xu, 2012 and others) showing the strong association with development of different cancer types including esophageal and cervical cancers.

We studied the following genetic markers: (1) deletion polymorphism of genes participating in second phase of xenobiotic detoxification – glutathione-*S*-transferases – *GSTM1* and *GSTT1*; (2) two types of single nucleotide polymorphism (SNP) of *XRCC1* (Arg194Trp and Arg399Gln), responsible for the repair of double strand DNA breaks; (3) SNP of *XRCC3* (Thr241Met), responsible for the repair of single strand DNA breaks; (4) SNP of gene regulating cell cycle and apoptosis – *TP53* (Arg72Pro); (5) SNP of cell cycle regulating gene cyclin D1 – *CCND1* (A870G).

## Materials and Methods

### Sampling

This “case-control” study was approved by the Ethics Committee of the Asfendiyarov Kazakh National Medical University (Almaty, Kazakhstan). The material was collected in the Kazakh Research Institute of Oncology and Radiology (Almaty, Kazakhstan) by approbation of the patients. We examined clinical material (blood, buccal smears, biopsy materials, cervical smears) obtained from 115 esophageal cancer patients and 207 cervical cancer patients. The control groups of healthy individuals (100 and 160 respectively) were selected according to the ethnic background and age of the esophagus and cervical cancer patients. Detailed questionnaires and informed consents were filled prior collection of samples. The clinical diagnosis of cancer patients was verified by the cytological or histological methods using biopsy materials.

### DNA isolation

DNA samples were extracted by standard phenol-chloroform method with modifications in lysis buffer composition (for blood samples: 0.2 M sodium acetate and 1% sodium dodecyl sulfate, pH 8.0; for tissue: 0.02 M ethylenediaminetetraacetic acid (EDTA); 0.02 M Tris-HCl, pH = 8.0; 0.16 M NaCl; 0.3% sodium dodecyl sulfate, 1 U of protease E). Water diluted DNA samples were used for all type of polymerase chain reaction (PCR).

### Genotyping by site-specific PCR amplification

The genotyping of *GSTM1* and *GSTT1* deletion polymorphisms was carried out by multiplex PCR amplification. The method of site-specific PCR amplification followed by restriction of amplified fragments was used for the genotyping of *XRCC1* Arg194Trp; *XRCC1* Arg399Gln, *XRCC3* Thr241Met, and *TP53* Arg72Pro SNPs. Twenty to one hundred nanogram of target DNA was amplified in total volume of 20 μl of PCR mixtures using amplifier “Mastercycler” (Eppendorf). PCR mixture contains 15 pM of each specific primer, 10 mM of each dNTP, 2 μl of 10× PCR buffer (10 mM KCl, 100 mM Tris-HCl, pH 9.0), and 0.5 U of Taq-polymerase (Sigma-Aldrich). The 1.4% agarose gel electrophoresis and Lambda/*Hin*dIII DNA marker (Sigma-Aldrich) were used for detection of the amplified DNA fragments length. The PCR products were digested at 37°C for 8–16 h with 1–3 U corresponding restriction enzymes (Fermentas, Lithuania). Restriction products were analyzed using 3% agarose MetaPhor (Lonza) gel. The PCR details and corresponding references are represented in Table 1.

**Table 1:** **The site-specific PCR amplification protocols**.

Genes	Primers for PCR	PCR conditions	The length of amplified fragments (bp)	Restriction enzyme	Restricted products length and corresponding genotype	Reference
*GSTM1*	s 5′-GAACTCCCTGAA AAGCTAAAG C-3′, as 5′-GTTGGGCTCAAATATACGGTGG-3′	Initiation denaturation step at 94°C for 5 min, followed by 35 cycles of 94°C for 2 min, 59°C for 1 min, 72°C for 1 min and final elongation step at 72°C for 10 min.	215	Not used	–	Abbas et al. (2004)
*GSTT1*	s 5′-TTCCTTACTGGTCC TCACATCTC-3′, as 5′-TCACCGGATCATGGCCAGCA-3′		480	Not used	–	
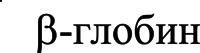 (as a internal control)	s 5′-CCACTTCATCCACGTTCACC-3′, as 5′-GAAGAGCCTAGGACAGGTAC-3′		268	Not used	–	
*XRCC1* Arg194Trp	s 5′-GCCCCGTCCCAGGTA-3′, as 5′-AGCCCCAAGACCCTTT-3′	Initiation denaturation step at 95°C for 2 min, followed by 40 cycles of 94°C for 15 s, 57°C for 45 s, 72°C for 45 s and final elongation step at 72°C for 5 min	490	*Pvu*II, 10 × Tango buffer	Arg/Arg – 490 bp; Arg/Trp – 490, 294, and 196 bp; Trp/Trp – 294 and 196 bp	Au et al. (2003)
*XRCC1* Arg399Gln	s 5′-CAAGTACAGCCAGGTCCTAG-3′, as 5′-CCTTCCCTCATCTGGAGTAC-3′	Initiation denaturation step at 95°C for 2 min, followed by 40 cycles of 94°C for 15 s, 55°C for 30 s, 72°C for 45 s and final elongation step at 72°C for 5 min	248	*Bcn1*, 10 × Tango buffer	Arg/Arg – 159 and 89 bp; Arg/Gln – 248, 159, and 89 bp; Gln/Gln – 248 bp	Au et al. (2003)
*XRCC3* Trp241Met	s 5′-GCCTGGTGGTCATCGACTC-3′, as 5′-ACAGGGCTCTGGAAGGCACTGCTCAGC TCACGCACC-3′	Initiation denaturation step at 94°C for 3 min, followed by 35 cycles of 95°C for 1 min, 60°C for 1 min, 72°Cfor 1 min and final elongation step at 72°C for 5 min	136	*Nco1*, 10 × Tango buffer	Trp/Trp – 136 bp, Trp/Met – 136, 97, and 39 bp; Met/Met – 97 and 39 bp	Au et al. (2003)
*TP53* Arg72Pro	s 5′-TGAGGACCTGGTCCTCTGAC-3′, as 5′-AGAGGAATCCCAAAGTTCCA-3′	Initiation denaturation step at 94°C for 2 min, followed by 35 cycles of 94°C for 30 s, 54°C for 30 s, 72°C for 30 s and final elongation step at 72°C for 5 min	412	*Bsh*1236I *(Acc II)*, 10 × r buffer	Arg/Arg – 252 and 160 bp; Arg/Pro – 412, 252 and 160 bp; Pro/Pro – 412 bp	Lu et al. (2004)

### Genotyping by direct sequencing

The method of direct sequencing was applied for genotyping of *CCND1* A870G polymorphism for all DNA samples. This method also was used for some DNA samples representing esophageal cancer in the case of determination of *TP53* Arg72Pro polymorphism. Previously we performed the PCR amplification of the gene fragments which contain the studied polymorphic sites: for *CCND1* (281 bp) and for *TP53* gene (141 bp). The PCR was carried out in a total volume of 25 μl reaction mixture containing 50 ng of target DNA, 0.625 U of ExTaq™ HS enzyme (TaKaRa Biotechnology, Japan), 0.2 mM of each dNTP, 2.5 μl of 10× PCR buffer (100 mM KCl, 0.1 mM EDTA, 1 mM DTT, 0.5% Tween 20, 0.5% Non-idet P-40, 50% glycerol, 20 mM Tris-HCl, pH 8.0), and 200 nM of each primer (for the *CCND1* gene – s 5′-CGG GCC GCT TGC TCA GAG-3′ and as 5′-AAG GCT GCC TGG GAC ATC ACC-3′, for *TP53*: gene – s 5′-CGT CCC AAG CAA TGG ATG ATT-3′ and as 5′-CCG GTG TAG GAG CTG CTG G-3′). The PCR amplification conditions consisted of initial denaturation step at 95°C for 5 min, followed by 35 cycles of 94°C for 30 s, 58°C for 30 s (for the *CCND1* gene), or 61°C for 30 s (for the *TP53* gene), 72°C for 30 s, and final elongation step at 72°C for 10 min. The 1.5% agarose gel electrophoresis and Lambda/*Hin*dIII DNA marker (Sigma-Aldrich) were used for detection of the amplified DNA fragments length. Amplified DNA fragments was purified from residues of PCR reaction mixture using ExoSAP-IT^®^ (GE Healthcare, USA). To 5 μl of PCR product we added 1 μl of ExoSAP-IT^®^ and incubated at 37°C for 40 min, at 80°C for 20 min, and at 4°C for 10 min. Sequencing of PCR products was carried out using the BigDye^®^ Terminator v3.1 kit (Applied Biosystems) in accordance with standard protocol^1^. Sequence-amplification was carried out in the total volume 20 μl reaction mixture containing 3 μl of PCR product, 4 μl of 5 × BigDye^®^ buffer, 0.5 μl ready mix BigDye^®^ Terminator v3.1, 3.2 pM of specific forward primer (for gene *CCND1*: s 5′-CGG GCC GCT TGC TCA GAG-3′and for the gene *TP53*: s 5′-CGT CCC AAG CAA TGG ATG ATT-3′).

Sequence-PCR conditions were the same for *CCND1* and *TP53* genes: initial step at 94°C for 2 min, followed by 30 cycles of 96°C for 30 s, 60°C for 4 min, and final elongation step at 4°C for 10 min. Sequence-PCR products were filtered using Sephadex™ G-50 (Amersham Biosciences) by centrifugation at 1000 × *g* for 3 min. Then 20 μl of formamide was added to the purified sequence-PCR product and denatured at 95°C for 2–3 min followed by cooling on ice. Genotyping of the products obtained sequencing was performed using capillary analyzer ABI PRISM^®^ 3130 (Applied Biosystems). Data analysis was carried out using the program FinchTV (Geospiza, USA).

### Genotyping by the TaqMan allelic discrimination method

Genotyping of *TP53* Arg72Pro polymorphism of some esophageal cancer samples was also carried out by the TaqMan allelic discrimination method. We used the specially synthesized probes (Applied Biosystems) containing the fluorescent reporter dye on the 5′-end and the quencher dye on the 3′-end. The probe complementary to the Pro72 allele was labeled by FAM™ dye, the probe complementary to the Arg72 allele – by VIC™ dye. Amplification of *TP53* gene fragment (141 bp) containing the polymorphic site Arg72Pro was performed by real-time PCR using a thermocycler iCycler iQ5 (Bio-Rad). The amplification was carried out in a total volume of 25 μl reaction mixture containing 50 ng of target DNA, 12.5 μl of universal PCR-mix TaqMan^®^ (Applied Biosystems), 10 μM of each primers (s 5′-CGT CCC AAG CAA TGG ATG ATT-3′ and as 5′-CCG GTG TAG GAG CTG CTG G-3′), and 14 μM of each probe [for the Pro72 (FAM) allele – 5′-CTC CCC GCG TGG CCC C-3′ and for the Arg72 (VIC) allele – 5′-CTC CCC CCG TGG CCC C-3′]. The real-time PCR conditions consisted of initiation denaturation step at 50°C for 2 min and 95°C for 10 min followed by 35 cycles of 95°C for 15 s and 61°C for 1 min, and final step at 4°C for 10 min. Fluorescence end point detection and analysis of data were performed using ABI PRISM^®^ 7700 Sequence Detection System (Applied Biosystems) and supporting Software.

### Statistical analysis

The allele frequencies was calculated in accordance with standard Hardy–Weinberg equilibrium: *p*^2^ + 2*pq* + *q*^2^ = 1, where *p* – the frequency of allele 1 and *q* – the frequency of allele 2.

To estimate the relative risk of cancer development we use the method of odd ratio (OR) calculation for case-control epidemiologic study taking into account the dominant and recessive models (Jedrychowski and Maugeri, 2000). In general model OR is calculated for each genotype (11, 12, 22) separately. According to the dominant model OR is calculated comparing the normal homozygous (11) versus combination of mutant homozygous (22) with heterozygous (12). The recessive model: combination of normal homozygous (11) with heterozygous (12) versus mutant homozygous (22). An approximate index of relative risk, or OR, is the ratio of disease development chances among persons, exposed and did not exposed to some factor (in our case this is genotype). OR, close to 1, indicates the absence of the genotype effect on disease development. More than 1 OR value indicates the influence of genotype on development of disease, and less than 1 OR value indicates a positive effect of genotype on health. OR may be calculated by the following formula:

(1) OR=ad/bc,

where

a – The number of persons in case group (patients with disease) having genotype 1;
b – the number of persons in case group having genotype 2;
c – the number of persons in control group (healthy persons) having genotype 1;
d – the number of persons in control group having genotype 2.

The estimates of 95% confidence intervals (CI) can be computed from the following formula:

$$\begin{array}{l} \text{Lower }95\%\text{ CI}=\exp\left(\ln\text{ OR}-1.96\sqrt{\left(1/a+1/b+1/c+1/d\right)}\right)\\ \text{Upper }95\%\text{ CI}=\exp\left(\ln\text{ OR}+1.96\sqrt{\left(1/a+1/b+1/c+1/d\right)}\right) \end{array}$$

To verify the significance (*p*-values) of the observed differences between case and control groups, we performed the standard χ^2^ test. An alpha error (*P*) of less than 0.05 was used as the criterion of significance.

All statistical analysis of the obtained data was performed using GraphPad InStat™ Software (V. 2.04. Ralf Stahlman, Purdue University) and “Case-Control Study Estimating Calculator” from TAPOTILI company (Laboratory of Molecular Diagnostics and Genomic Dactiloscopy of “GosNII Genetika” State Scientific Centre of Russian Federation^2^).

## Results

### A distinct polymorphism associates with esophageal cancer

All 115 patients representing the esophageal cancer group were diagnosed as the squamous-cell carcinoma cancer type. In this group, the high differentiated carcinoma was detected in 8 patients, the moderately differentiated carcinoma in 48 patients, and the low-grade differentiated carcinoma in 59 patients. The control cohort represented the healthy people without any noticeable pathologies was matched to case cohort by the age, sex, and ethnicity, and smoking habit (Table 2).

**Table 2:** **The correspondence of the esophageal cancer case and control cohorts by age, ethnicity, sex, and smoking habit**.

Cohort	Years of birth	Nationality, persons (%)		Sex, persons (%)		Smoking habit, persons (%)	Total, persons
		Kazakh	Russian	Male	Female	
Case	1920–1977	102 (88.69)	13 (11.31)	62 (53.91)	53 (46.09)	30 (26.08)	115
Control	1921–1976	89 (89.00)	11 (11.00)	54 (54.00)	46 (46.00)	26 (26.00)	100

Genotyping of the candidate genes (deletions *GSTM1* and *GSTT1*, *XRCC1* Arg194Trp and Arg399Gln, *XRCC3* Thr241Met, *TP53* Arg72Pro, and *CCND1* A870G) was performed for the case and control cohorts. Frequencies of the allele variants are shown in Table 3.

**Table 3:** **The frequencies of alleles of candidate genes in control and case cohorts for esophageal cancer**.

Polymorphism	The allele variant	The frequency of allele	
		Control cohort	Case cohort
*GSTT1*	Functional (+)	0.365	0.178
	Deletion (−)	0.635	0.822
*GSTM1*	Functional (+)	0.510	0.204
	Deletion (−)	0.490	0.796
*XRCC1* Arg194Trp	194Arg	0.855	0.852
	194Trp	0.145	0.148
*XRCC1* Arg399Gln	399Arg	0.725	0.657
	399Gln	0.275	0.343
*XRCC3* Trp241Met	241Trp	0.815	0.822
	241Met	0.185	0.178
*TP53* Arg72Pro	72Arg	0.760	0.657
	72Pro	0.240	0.343
*CCND1* A870G	870A	0.450	0.404
	870G	0.550	0.596

Genotyping of the candidate genes shows no contradictions with the Hardy–Weinberg equilibrium. However, the frequencies of allele variants differ between the control group and the group representing esophageal cancer patients. We also performed the statistical analysis of association between genetic polymorphism and development of esophageal cancer. The statistical powers of the general, dominant, and recessive models were evaluated for each type of polymorphism. Table 4 shows the adjusted association of candidate gene polymorphisms, which calculated by general model of inheritance for each genotype separately.

**Table 4:** **Association between genetic polymorphism and development of esophageal cancer**.

Type of polymorphism	Genotype	Esophageal cancer, persons (%)	Control, persons (%)	Odds ratio (OR)	Confidence interval (CI), (95%)	χ2	*p*
*GSTT1*	+/+	5 (4.35)	13 (13.00)	0.30	0.10–0.89	18.66	<0.0001
	±	31 (26.96)	47 (47.00)	0.42	0.24–0.74	
	−/−	79 (68.69)	40 (40.00)	3.29	1.88–5.77	
*GSTM1*	+/+	4 (3.48)	26 (26.00)	0.10	0.03–0.31	40.64	<0.0001
	±	39 (33.91)	50 (50.00)	0.51	0.30–0.89	
	−/−	72 (62.61)	24 (24.00)	5.30	2.93–9.61	
*XRCC1* Arg194Trp	Arg/Arg	85 (73.91)	72 (72.00)	1.11	0.60–2.01	1.86	0.39
	Arg/Trp	26 (22.61)	27 (27.00)	0.79	0.42–1.47	
	Trp/Trp	4 (3.48)	1 (1.00)	3.57	0.39–32.46	
*XRCC1* Arg399Gln	Arg/Arg	47 (40.87)	49 (49.00)	0.72	0.42–1.23	3.24	0.2
	Arg/Gln	57 (49.57)	47 (47.00)	1.11	0.65–1.90	
	Gln/Gln	11 (9.56)	4 (4.00)	2.54	0.78–8.24	
*XRCC3* Thr 241Met	Trp/Trp	82 (71.30)	64 (64.00)	1.40	0.79–2.48	8.32	0.02
	Trp/Met	25 (21.74)	35 (35.00)	0.52	0.28–0.94	
	Met/Met	8 (6.96)	1 (1.0)	7.40	0.91–60.25	
*TP53* Arg72Pro	Arg/Arg	51 (44.38)	57 (57.00)	0.60	0.35–1.03	5.71	0.06
	Arg/Pro	49 (42.61)	38 (38.00)	1.21	0.70–2.09	
	Pro/Pro	15 (13.04)	5 (5.00)	2.85	1.00–8.15	
*CCND1* A870G	G/G	22 (19.13)	28 (28.00)	0.61	0.32–1.15	10.87	0.004
	G/A	49 (42.61)	54 (54.00)	0.63	0.37–1.08	
	A/A	44 (38.26)	18 (18.00)	2.82	1.50–5.32	

The prevalence of *GST*-deletions (−/−, “null” – genotype) was observed in esophageal cancer cases cohort. Our data show significant association of “null” *GST*-genotypes (−/−) with susceptibility to esophageal cancer for *GSTM1* (OR = 5.30) and *GSTT1* (OR = 3.29) genes. These findings are also confirmed by the dominant (+/+ versus combination of ±and −/− genotypes) and recessive models (−/− versus combination of +/+ and ±genotypes) of OR calculation. Thus, according to the dominant model the risk of esophageal cancer development was significantly higher for the following combinations of genotypes: *GSTM1* (± and −/−) (OR = 9.75, *p* < 0.0001); and *GSTT1* (± and −/−) (OR = 3.29, *p* = 0.02). The recessive model corresponds to the results of general model of inheritance: for the *GSTM1* −/− (OR = 5.30, *p* < 0.0001) and *GSTT1* −/− (OR = 3.29, *p* < 0.0001). The presence of the functional allele variants of *GSTM1* and *GSTT1* genes in homozygous states shows a strong protective effect (for *GSTM1* +/+ genotype – OR = 0.10 and for *GSTT1* +/+ genotype – OR = 0.30).

The difference of *XRCC1* Arg194Trp polymorphism genotypes distribution between control and esophageal case cohorts was not significant. The *XRCC1* Trp194Trp homozygote did not show a statistically reliable association with esophageal cancer (OR = 3.57, *p* = 0.39). Application of the dominant and recessive model of OR calculation did not reveal any significant risk. We have noticed a similar relationship following analysis of the *XRCC1* polymorphism – Arg399Gln that indicated OR values [OR = 2.54, *p* = 0.2 (general model of inheritance); OR = 1.39, *p* = 0.23; dominant model] were not statistically significant. On the contrary, analysis of the homozygote genotype *XRCC3* Met241Met (OR = 7.40, *p* = 0.02) detected a strong linkage to the esophageal cancer progression. This finding has been supported by the dominant model. Instead, the protective effect (OR = 0.52, *p* = 0.02) was observed with the heterozygous genotype *XRCC3* Trp241Met.

Comparison of the *TP53* Arg72Pro genotypes distribution between control and case cohorts shows the prevalence of Pro/Pro homozygous (13 versus 5%) and Arg/Pro heterozygous (43 versus 38%) among esophageal cancer patients. The association of *TP53* Pro72Pro genotype with susceptibility to esophageal cancer has been analyzed by the total model of inheritance – OR = 2.85, *p* = 0.06. The presence of Arg in 72 codon of *TP53* gene reduced the risk: OR = 1.66, *p* = 0.06 (dominant model – Pro72Pro in combination with Arg72Pro); for Arg72Pro genotype – OR = 1.21, *p* = 0.06. Whereas, a strong protective effect has been observed for the Arg72Arg genotype – OR = 0.60, *p* = 0.06.

Substantial prevalence of *CCND1* A870A homozygous has been detected in the esophageal cancer case cohort (38 versus 18% in control). The *CCND1* A870A genotype is statistically reliable to determine its susceptibility to esophageal cancer (OR = 2.82, *p* = 0.004). Combination with *CCND1* G870A genotype (dominant model) reduces the risk: OR = 1.64, *p* = 0.12. The G870G genotype demonstrates strong protective effect: OR = 0.61, *p* = 0.004.

### Association of genomic polymorphism with development of cervical cancer

All 217 women representing cervical cancer case cohort were cytologically or histologically examined for cancer type. Squamous-cell carcinoma (cancer *in situ*) is the predominant histotype in selected cohort. Within this cohort, 15 patients were at the stage I, 167 patients were at the stage II, 26 patients were at the stage III (invasive), and 9 patients were at the stage IV (invasive, metastatic).

The control cohort of healthy women was selected taking into account the personal data of patients suffering from cervical cancer. The age, ethnicity, and smoking habit data of cervical cancer case and control groups are represented in Table 5.

**Table 5:** **The correspondence of the cervical cancer case and control cohorts by age, ethnicity, and smoking habit**.

Cohort	Years of birth	Nationality, persons (%)		Smoking habit, persons (%)	Total, persons
		Kazakh	Russian	
Case	1945–1990	176 (81.11)	41 (18.89)	10 (4.61)	217
Control	1942–1987	128 (80.00)	32 (20.00)	8 (5.00)	160

DNA samples representing the cervical cancer case and control cohorts were genotyped for detection of different types of gene polymorphisms: deletions *GSTM1* and *GSTT1*, *XRCC1* Arg194Trp and Arg399Gln, *XRCC3* Thr241Met, *TP53* Arg72Pro, and *CCND1* A870G.

The genotyping results revealed that the distribution of genotypes in the control and case cohorts follows to Hardy–Weinberg equilibrium. Frequencies of allele variants are summarized in Table 6.

**Table 6:** **The frequencies of alleles of candidate genes in control and case cohorts for cervical cancer**.

Polymorphism	The allele variant	The frequency of allele	
		Control cohort	Case cohort
*GSTT1*	Functional (+)	0.544	0.230
	Deletion (−)	0.456	0.770
*GSTM1*	Functional (+)	0.850	0.677
	Deletion (−)	0.150	0.323
*XRCC1* Arg194Trp	194Arg	0.781	0.862
	194Trp	0.219	0.138
*XRCC1* Arg399Gln	399Arg	0.694	0.634
	399Gln	0.306	0.366
*XRCC3* Trp241Met	241Trp	0.875	0.776
	241Met	0.125	0.224
*TP53* Arg72Pro	72Arg	0.572	0.647
	72Pro	0.428	0.353
*CCND1* A870G	870A	0.500	0.491
	870G	0.500	0.509

Comparison of the candidate genes allele frequencies in cohorts of healthy women and women suffering from cervical cancer shows the differences including a prevalence of the *GST-*deletions and rare allele variant *XRCC3* 241Met in case cohort. The data of statistical analysis of associations between the studied gene polymorphisms and susceptibility to cervical cancer, which calculated for each genotype separately by general genetic model, are presented in Table 7.

**Table 7:** **Association between genetic polymorphism and development of cervical cancer**.

Type of polymorphism	Genotype	Cervical cancer, persons (%)	Control, persons (%)	Odds ratio (OR)	Confidence interval (CI), (95%)	χ2	*p*
*GSTT1*	+/+	12 (5.53)	57 (35.62)	0.11	0.05–0.21	67.15	0.00
	±	76 (35.02)	60 (37.50)	0.90	0.59–1.37	
	−/−	129 (59.45)	43 (26.88)	3.99	2.56–6.21	
*GSTM1*	+/+	108 (49.77)	116 (72.50)	0.38	0.24–0.58	25.31	<0.0001
	±	78 (35.94)	40 (25.00)	1.68	1.07–2.65	
	−/−	31 (14.29)	4 (2.5)	6.5	2.25–18.81	
*XRCC1* Arg194Trp	Arg/Arg	163 (75.12)	105 (65.63)	1.58	1.01–2.48	8.72	0.01
	Arg/Trp	48 (22.12)	40 (25.00)	0.85	0.53–1.38	
	Trp/Trp	6 (2.76)	15 (9.37)	0.27	0.10–0.73	
*XRCC1* Arg399Gln	Arg/Arg	78 (35.94)	66 (41.25)	0.80	0.53–1.22	7.24	0.03
	Arg/Gln	119 (54.84)	90 (56.25)	0.94	0.63–1.42	
	Gln/Gln	20 (9.22)	4 (2.50)	3.96	1.33–11.82	
*XRCC3* Trp241Met	Trp/Trp	140 (64.51)	124 (77.50)	0.53	0.33–0.84	10.28	0.006
	Trp/Met	57 (26.27)	32 (20.00)	1.43	0.87–2.33	
	Met/Met	20 (9.22)	4 (2.50)	3.96	1.33–11.82	
*TP53* Arg72Pro	Arg/Arg	85 (39.17)	49 (30.63)	1.46	0.95–2.25	5.15	0.08
	Arg/Pro	111 (51.15)	85 (53.12)	0.92	0.61–1.39	
	Pro/Pro	21 (9.68)	26 (16.25)	0.55	0.30–1.02	
*CCND1* A870G	G/G	54 (25.12)	41 (25.62)	0.97	0.61–1.56	0.09	0.96
	G/A	103 (47.91)	78 (48.75)	0.97	0.64–1.46	
	A/A	58 (26.97)	41 (25.63)	1.07	0.67–1.71	

Deletion of *GSTT1* in homozygous state (−/−) shows the significant association with susceptibility to cervical cancer (OR = 3.99, *p* = 0.0). *GSTT1* “null” genotype in combination with heterozygous genotype (−/− and ±) significantly increases the risk: in accordance with dominant model of OR calculation – OR = 9.45, *p* = 0.0. The *GSTM1* “null” genotype also shows strong association with development of cervical cancer (OR = 6.50, *p* < 0.0001). But the *GSTM1* functional allele presence in genotype reduces the risk. In accordance with dominant model for the combination of *GSTM1* genotypes (± and −/−) – OR = 2.66, *p* < 0.0001. The presence in genotype of the functional allele variants of *GSTM1* and *GSTT1* genes in homozygous states shows strong protective effect (for *GSTM1* +/+ genotype – OR = 0.38 and for *GSTT1* +/+ genotype – OR = 0.11).

The *XRCC1* 194Arg allele variant shows association with susceptibility to cervical cancer in homozygous (Arg194Arg) and heterozygous (Arg194Trp) states. According to general model of inheritance for Arg194Arg genotype – OR = 1.58, *p* = 0.01. According to the dominant model for combinations of genotypes (Arg194Arg and Arg194Trp) – OR = 3.64, *p* = 0.006.

Polymorphism of 399 codon of *XRCC1* gene also shows association with susceptibility to cervical cancer. For the homozygous genotype Gln399Gln – OR = 3.96, *p* = 0.03. The presence of 399Arg allele variant in genotype reduces the risk, but not statistically reliably: OR = 1.25, *p* = 0.90 (dominant model – Gln399Gln in combination with Arg399Gln). The protective effect of *XRCC1* Arg399Arg genotype is weakly expressed (OR = 0.80, *p* = 0.03).

Another DNA repair gene *XRCC3* demonstrates the strong association between Trp241Met polymorphism and susceptibility to cervical cancer. The risk is expressed for homozygotes Met241Met: OR = 3.96, *p* = 0.006. In combination with heterozygous genotypes (dominant model – Met241Met and Trp241Met versus Trp241Trp) the risk is significantly reduced: OR = 1.89, *p* = 0.007. The Trp241Trp genotype shows the statistically reliable protective effect: OR = 0.53, *p* = 0.006.

Analysis of *TP53* Arg72Pro polymorphism genotypes distribution in control and cervical cancer case cohorts shows that Arg72Arg genotype can increase risk of cervical cancer development (OR = 1.46, *p* = 0.08), but not significantly. The risk is increased (OR = 1.89, *p* = 0.06) in combination with heterozygous genotype (dominant model of inheritance – Arg72Arg and Arg72Pro versus Pro72Pro). *TP53* Pro72Pro genotype demonstrates the protective effect (OR = 0.55, *p* = 0.08).

The *CCND1* G870A polymorphism does not show significant differences in genotype distribution among healthy women and the cervical cancer patients. We also did not observe association of this variation with susceptibility to cervical cancer.

## Discussion

Aging is a complex process of functional decline and increased disease risk that has been resulted from accumulation of DNA mutations. Series of mutations in key regulatory genes are the main reason of cancer induction. Genes involved into the control of genome instability, DNA repair, cell cycle regulation, apoptosis, and such processes as xenobiotics detoxification, are the main candidate for aging and carcinogenesis. Mutations affecting the functions of these genes cause the range of abnormalities. Polymorphic variants of their DNA sequences can modify the functions of genes and predispose to the disease development in combination with other genetic and environmental factors.

The frequency of alleles of polymorphic genes is determined by natural selection. Many factors affect the genomic polymorphism spectrum in populations, such as geographical location, ethnicity, type of diet, habits, etc. The ethno-genetic status, age, the radiation background, and bad habits strongly influence on mutagenic processes.

Numerous of molecular epidemiological studies have been devoted to finding biomarkers of age-related diseases. However, the revealing of reliable association between polymorphic allele variant and susceptibility to disease depends on allele frequency in population. The high frequency of allele facilitates the identification of the association with high probability. In the case of rare allele the detection of association is more complicated, and it requires an increase of sample size.

Epidemiological studies require a careful selection of the control group for the research, especially for small sample sizes. The control cohort should correspond to case cohort on many parameters. Matching control can help to identify the reliable association between genetic polymorphism and risk of disease in the cases of small sample sizes or rare allele frequency.

The studied cohorts represent inhabitants of Almaty city (Kazakhstan). Radiation background in Almaty is not significant and this factor has not been considered in our study. However, smoking and age are known risk factors for many cancer types. Also, the case cohorts representing patients suffering from esophageal and cervical cancer are mixed by ethnicity. The majority of both case cohorts are Kazakhs (about 80%), but there are Russians too (about 20%). To minimize the effects of ethnicity, age, and smoking influence on the susceptibility to studied cancer types, we have selected the healthy control groups matched to the corresponding case groups (Tables 2 and 5).

The genotyping on candidate gene polymorphisms allowed us to determine all possible genotypes in the studied control and case cohorts in accordance with Hardy–Weinberg equilibrium.

Because these types of genetic polymorphisms were first studied for Kazakh populations, we compared the obtained frequency of allele variants in control cohorts with data presented in NCBI SNP database and literature (d’Errico et al., 1999; Ketterer et al., 2007; Gao et al., 2011). For the increasing of sample size we have combined both control cohorts because all investigated persons were healthy inhabitants of Almaty city. The integrated data presented in Table 8.

**Table 8:** **The comparison of rare allele frequencies of healthy inhabitants of Almaty city with earlier studied populations**.

Polymorphism	The rare allele variant	The frequency of allele		
		Healthy residents of Almaty city (260 persons)	Integrated data from different sources	
			Asian populations	European populations
*GSTT1*	Deletion	0.525	0.480–0.540	0.160–0.385
*GSTM1*	Deletion	0.281	0.490–0.540	0.420–0.540
*XRCC1* Arg194Trp	194Trp	0.190	0.239–0.289	0.092–0.093
*XRCC1* Arg399Gln	399Gln	0.294	0.274–0.279	0.303
*XRCC3* Trp241Met	241Met	0.150	0.000–0.148	0.000–0.417
*TP53* Arg72Pro	72Pro	0.356	0.409–0.511	0.233
*CCND1* A870G	870G	0.481	0.456–0.656	0.475–0.483

The frequencies of *GSTT1* deletions in healthy residents of Almaty city (0.525) are more similar to Asians (0.80–0.540). The *GSTM1* deletions are widely distributed among Asian (0.490–0.540) and European (0.420–0.540) peoples with similar rates. But in our study the frequency of *GSTT1* deletions was low: 0.281. The low frequencies of *GSTT1* deletions have been suggested for African populations (0.160–0.360) (d’Errico et al., 1999; Ketterer et al., 2007; Gao et al., 2011).

The data on frequencies of rare *XRCC1* 399Gln, *XRCC3* 241Met, and *CCND1* 870G alleles in healthy residents of Almaty city do not contradict to experimental data obtained from the analysis of most Asian and European populations (Table 8).

The rates of *XRCC1* 194Trp (0.190) and *TP53* 72Pro (0.356) alleles do not correspond to the populations from Europe and Asia (Table 8). One of the possible explanations is the mixed ethnic composition of Almaty city residents (Tables 2 and 5): 80% Kazakh (Asians) and 20% Russian (Europeans). Also it should be noted that most of studied populations from Asia, represented in NCBI SNP database and other sources (Chinese, Japanese, Malaysian, etc.), were distinct from Kazakh population.

Identified associations between candidate genes polymorphism and esophageal and cervical cancer are not surprising. Glutathione *S*-transferases (*GST*s), a multigene family of phase II metabolic enzymes, are active in the detoxification of a wide variety of potentially toxic and carcinogenic substances by conjugating them to glutathione. Deletions of *GST*-genes are associated with susceptibility to many cancer types. The previous study (Tan et al., 2000; Gao et al., 2002; Lu et al., 2006; Liu and Xu, 2012) reported that deletions of *GSTT1* and *GSTM1* genes play a significant role in development of esophageal or cervical cancers. Most of these studies were carried on Chinese and Caucasian populations. Association of *GSTT1* and *GSTM1* deletions with esophageal and cervical cancer susceptibility is supported by data obtained by studying populations from India, Korea, Turkey, Great Britain, Italy, USA, and other countries (Ketterer et al., 2007; Gao et al., 2011; Zhang et al., 2012). Our results demonstrate that *GSTT* and *GSTM* “null” genotypes are strongly associated with susceptibility to esophageal and cervical cancers in population from Kazakhstan (Almaty city).

But some data obtained on different populations from Japan, Brazil, Thailand, and Greece were distinct from our results (Morita et al., 1997; Rossini et al., 2007; Gao et al., 2011; Zhang et al., 2012). This fact can be explained by the insufficient knowledge about influence of *GST*-deletions on development of esophageal and cervical cancer or the distinct ethnic backgrounds or accounting the risk related factors, such as smoking, chemotherapy, or radiation therapy.

There are many opinions about influence of *XRCC1* (X-ray repair complementing defective repair in Chinese hamster cells 1) and *XRCC3* (X-ray repair complementing defective repair in Chinese hamster cells 3) genes on different cancer types. These genes participate in excision repair of bases and repair of single and double strand breaks. The previous studies also point out to the relation of *XRCC1* (Arg399Gln, Arg194Trp) and *XRCC3* Trp241Met polymorphisms with colorectal cancer, skin cancer, lung cancer (Cui et al., 2012; Zhang et al., 2012), and others.

There are data confirming the participation of *XRCC1*-genes polymorphism to cervical cancer (Li et al., 2012). Interestingly, that Barbisan et al. (2011) have made a conclusion, that Arg194Trp polymorphism may be associated with cervical cancer risk, Arg399Gln polymorphism might be a low-penetrant risk factor for cervical cancer only at Asians. The meta-analysis of 16 studies (Li et al., 2012) found out that there were no obvious associations of *XRCC1* Arg399Gln polymorphism with cervical cancer risk. But in the subgroup analyses by ethnicity/country, a significantly increased risk was observed among Asian, especially among Chinese. The study of one Chinese population (Yu et al., 2004) shows the strong association between *XRCC1* Gln399Gln genotype and squamous-cell carcinoma of esophagus, and the smoking people have 4.2-fold increased risk in comparison with not smoking persons.

We found out that *XRCC1* Arg194Trp polymorphism had been associated with esophageal and cervical cancer in Kazakhstan population, but in different manner. *XRCC1* Trp194Trp genotype was associated with susceptibility to esophageal cancer, and *XRCC1* Arg194Arg genotype – with cervical cancer. Interestingly, the data of meta-analysis revealed the protective effect of the *XRCC1* 194Trp allele for tobacco-related types of cancer, which was compatible with the evidence of lower mutagen sensitivity for this allele (Rayjean et al., 2005). Possibly this protective effect of 194Trp allele is related not only tobacco, but also other toxic influences, such as drug and contraceptive treatment. Our research revealed the strong protective effect of *XRCC1* 194Trp allele in cervical cancer patients.

Regarding the *XRCC1* Arg399Gln polymorphism our results show the evidence of associations between *XRCC1* Gln399Gln genotype carriers and increased risk of cervical and esophageal cancer development, which is confirmed by other studies (Yu et al., 2004).

Published data on the relationship of *XRCC3* Trp241Met polymorphism with cancer risk are inconsistent (Au et al., 2003; Konstantinos and Theodoros, 2010; Settheetham-Ishida et al., 2011). However, the most studies show the association of *XRCC3* 241Met allele. But *XRCC3* 241Met allele did not increase the risk of cervical cancer development in the Chinese population (He et al., 2008) and among Thai women (Settheetham-Ishida et al., 2011). Our data demonstrate the strong association between *XRCC3* Met241Met genotype and expressed risk of susceptibility to both cervical and esophageal cancer in Kazakhstan populations.

Mutations and polymorphisms of cell cycle regulating genes (*CCND1* and *TP53*) can play the main role in many types of cancer. Proto-oncogene cyclin D1 is an activator of CDK kinases, whose activity is required for cell cycle G1/S transition. This protein has been shown to interact with tumor suppressor protein Rb and the expression of this gene is regulated positively by Rb. One meta-analysis (Chen et al., 2012) exhibited the statistically significant association between *CCND1* G870A polymorphism and a risk for cancers of the digestive tract, including esophageal cancer (Zhang et al., 2003; Cescon et al., 2009). Another meta-analysis suggested that this polymorphism has no significant association with esophageal cancer risk in the Caucasian or the Asian populations (He et al., 2013). There are no substantial data confirming the correlation of this type of polymorphism with cervical cancer. In our study we have shown that *CCND1* A870A genotype associates with susceptibility to esophageal cancer, but not to cervical cancer.

Polymorphism of *TP53* Arg72Pro can play dual role in cancer development (Francisco et al., 2010). On the one side, protein product of 72Arg allele more effectively induces apoptosis (Dumont et al., 2003). On the other side, 72Pro allele variant provide longevity of being in cell cycle G1-phase in which DNA repair processes are active (Sullivan et al., 2004). Also it was established, that oncoprotein E6 coding by viruses HPV-18 and HPV-16, can interact with p53 protein inducing its degradation. And 72Arg allele faster degradates E6 than 72 Pro (Storey et al., 1998; Tada et al., 2001). Further investigations show contradictive results. Thus, women from Taiwan, Thailand, Korea, Japan, China, and Hong-Kong show no association between *TP53* 72Arg/Pro polymorphism and HPV-associated and HPV-non-associated cervical cancer (Nishikawa et al., 2000; Settheetham-Ishida et al., 2004; Wu et al., 2004 and others). The study of women from India, Brazil, Chili, Peru, and women from Africa show this association (de Araujo and Villa, 2003; Ojeda et al., 2003; Mitra et al., 2005). Study of women in Greece, Holland, and Hungary revealed this positive association (Madeleine et al., 2000; Habbous et al., 2012 and others). And also there are evidences of influence of *TP53* Arg72Pro on development of esophageal cancer (Cescon et al., 2009; Ma et al., 2012). We find out that *TP53* 72Pro allele associates with susceptibility to cervical cancer and 72Arg allele shows strong association with esophageal cancer development.

A large number of molecular epidemiologic studies have been performed to evaluate the role of polymorphisms of *GST-*, *XRCC-*, *TP53*, and *CCND1* genes in various neoplasms. The results of these studies obtained on different populations can be contradictory. The evidence of an association between some rare allele variant and risk of disease can be achieved by the quantitative analysis of available publications – meta-analysis (Yin et al., 2009; Gao et al., 2011; Jiang et al., 2011; Chen et al., 2012; Cui et al., 2012; Li et al., 2012; Zhang et al., 2012; He et al., 2013).

Studies investigating the combined effect of *GST*-deletions, *XRCC1* (Arg194Trp and Arg399Gln), *XRCC3* (Thr241Met), *TP53* (Arg72Pro), and *CCND1* (A870G) will be very important for further evaluate the role of these polymorphism in different cancers. Data of association between seven genetic polymorphism types and two types of age-related cancers obtained on unstudied populations from Kazakhstan can be substantial input for meta-analysis. It is required for understanding the role of studied polymorphisms in the development of age-related pathologies in populations from Eurasia. And also the research results have a high practical significance.

Conducted research allowed to determine the panels of genetic markers of predisposition to the development:

1. Esophageal cancer – deletions of *GSTT1* (OR = 3.29) and *GSTM1* (OR = 5.30) genes; *XRCC3* Met241Met (OR = 7.40); *TP53* Pro72Pro (OR = 2.85), *CCND1* A870A (OR = 2.82).
2. Cervical cancer – deletions of *GSTT1* (OR = 3.99) and *GSTM1* (OR = 6.50) genes; *XRCC1* Arg194Arg (OR = 1.58); *XRCC1* Gln399Gln (OR = 3.83), *XRCC3* Met241Met (OR = 2.84), and *TP53* Arg72Arg (OR = 3.96).

These results are statistically reliable and will be used for developing of test-kits for the defining susceptibility to esophageal and cervical cancers. The introduction of these tests to the screening programs will allow to develop the large-scale preventive measures and will have an impact on reducing cancer incidence and mortality in Kazakhstan, helping to extend the qualitative longevity.

## Conflict of Interest Statement

The authors declare that the research was conducted in the absence of any commercial or financial relationships that could be construed as a potential conflict of interest.

## Footnotes

^1^http://www.appliedbiosystems.com

^2^http://www.tapotili.ru
